# Fungal glyceraldehyde 3-phosphate dehydrogenase GpdC maintains glycolytic mechanism against reactive nitrogen stress-induced damage

**DOI:** 10.3389/fmicb.2024.1475567

**Published:** 2024-10-11

**Authors:** Chihiro Kadooka, Nozomi Katsuki, Shunsuke Masuo, Saito Kojima, Madoka Amahisa, Kouta Suzuki, Yuki Doi, Norio Takeshita, Naoki Takaya

**Affiliations:** Faculty of Life and Environmental Sciences, University of Tsukuba, Ibaraki, Japan

**Keywords:** nitric oxide, nicotinamide adenine dinucleotide phosphate, ethanol fermentation, glycolysis, *Aspergillus nidulans*

## Abstract

Highly reactive nitrogen species (RNS) damage proteins, lipids, and nucleotides, and induce disordered intracellular metabolism. Microorganisms that respond to and defend against RNS include fungal pathogens that invade host tissues. However, the full picture of their mechanisms remains unknown. We identified a novel glyceraldehyde 3-phosphate dehydrogenase (GAPDH) isozyme (GpdC) in the fungus *Aspergillus nidulans*. This isozyme preferred NADP^+^, which was unlike glycolytic GpdA that uses NAD^+^ as a cofactor. Exogenous RNS induced expression of the encoding *gpdC* gene, which when disrupted, decreased intracellular GAPDH activity, mycelial proliferation, and ethanol fermentation under RNS stress. Under these conditions, fungal growth requires glucose instead of non-fermentable carbon sources, and intact pyruvate decarboxylase (*pdcA*) and alcohol dehydrogenase (*alcC*) genes indicated that fungal metabolism shifts from respiratory to glycolytic and ethanolic fermentation. These results indicated that GpdC is an alternative GAPDH isozyme that facilitates NADP^+^-dependent glycolysis and energy conservation, which constitutes a fungal mechanism of stress tolerance via metabolic adaptation.

## Introduction

Reactive nitrogen species (RNS), especially nitric oxide (NO), play diverse roles in various biological mechanisms including metabolic regulation, signal transduction, and energy conservation (Andrabi et al., 2023). Mammalian NO synthases oxidize arginine to produce NO, and transduce cellular signals (Förstermann and Sessa, 2012; Crane et al., 2010). Plants produce NO from nitrate via nitrate reductase (Yamasaki, 2000; Tiso et al., 2012). Nitric oxide generated from nitrite by nitrite reductases (Zumft et al., 1994; Shoun et al., 1992, Kim et al., 2010) is an intermediate of bacterial and fungal denitrification mechanisms. Bacterial respiratory NO reductases, and fungal cytochrome P450-type NO reductase P450nor participate in these mechanisms by reducing NO to gaseous nitrous oxide (Takaya and Shoun, 2000; Shoun et al., 2012). Fungi and plants assimilate nitrate via nitrite, which is a source of nitrosonium cation (NO^+^) RNS, especially under acidic conditions (Takaya and Shoun, 2000).

Besides playing physiologically important roles, highly reactive RNS are detoxified by organisms to prevent damage to cellular components. Fungal and bacterial flavohemoglobins oxidize NO to nitrate (Frey et al., 2002; Bonamore and Boffi, 2008; Forrester and Foster, 2012). *S*-nitrosoglutathione (GSNO) is associated with glutathione and NO metabolism, and GSNO reductase reduces GSNO back to glutathione (Zhou et al., 2016; Liu et al., 2001). Filamentous fungi produce unique tolerating mechanisms in response to RNS. The *Aspergillus nidulans* nitrosothionein-thioredoxin system detoxifies NO (Zhou et al., 2013). A similar function has recently been identified in yeast metallothionein (Yoshikawa et al., 2023). Furthermore, 2,5-diamino-6-(5-phospho-D-ribosylamino)pyrimidin-4(3*H*)-one is an intermediate of riboflavin synthesis in the yeast *Saccharomyces cerevisiae*, and it scavenges NO (Anam et al., 2020). Amino acid-biosynthesis mechanisms regulated in the presence of RNS govern metabolic adaptation for fungal survival (Amahisa et al., 2024). These and as yet unidentified fungal tolerating mechanisms of RNS are potential targets of antifungal agents because filamentous fungi, especially those in the phylum Ascomycota, which include human pathogens, defend against NO generated by infected hosts (Bogdan, 2001; Martínez-Medina et al., 2019).

Glyceraldehyde 3-phosphate (GAP) dehydrogenase (GAPDH; phosphorylating; E C. 1.2.1.12) catalyzes NAD^+^-dependent redox-coupled phosphorylation of GAP, and it is a key enzyme in glycolytic metabolism (Michels et al., 1996; Weber and Bernhard, 1982). It is also a target of reactive oxygen species (ROS) and RNS, the latter of which post-translationally modifies its thiol moiety at catalytic cysteine to disulfide, *S*-sulfenyl, *S*-sulfhydration, *S*-glutathione and *S*-nitrosation (Hara et al., 2005; Mustafa et al., 2009; Grant et al., 1999; Tsuchiya et al., 2018). These modifications inactivate GAPDH and regulate signal transduction, transcriptional regulation, and direct the cell fate to either apoptosis or programmed-cell death according to cellular redox status (Hara et al., 2006; Tossounian et al., 2020). Most bacterial and mammalian genomes encode only GAPDH, whereas fungal and plant genomes encode many GAPDH izozymes. The model plant *Arabidopsis thaliana* has seven genes that encode cytosolic NAD^+^-dependent GAPDH and chloroplast NADP^+^-dependent GAPDH (Martin and Cerff, 1986; Hildebrandt et al., 2015). Fungi produce glycolytic NAD^+^-dependent GAPDH encoded by *gpdA*, according to the nomenclature of the genus *Aspergillus* (Flipphi et al., 2009), under glycolysis. Functions other than glycolysis and post-translational modification of the fungal GpdA remain uncharacterized. Another GAPDH ortholog *gpdB* is distributed in most, but not all *Aspergillus* species. The *Aspergillus oryzae* GAPDH isozyme encoded by *gpdB* is resistant to a GAPDH inhibitor of heptelidic acid (Shinohara et al., 2019). The gene is synonymous with *hepG* that resides in the *hep* gene cluster for biosynthesizing the secondary metabolite heptelidic acid, suggesting its function in fungi that produce this antibiotic. The *gpdC* genes are more distantly related to *gpdA* and *gpdB*, and are predicted in *Aspergillus* genomes (Flipphi et al., 2009), but their physiological functions remain unclear.

This study identified a novel *A. nidulans* mechanism of RNS tolerance mediated by the GAPDH ortholog *gpdC*, the transcriptional level of which was increased by exposure to RNS. Analyzing its gene disruptant indicated that GpdC activity is important for glycolysis, ethanol fermentation mechanisms, and cell growth under RNS stress. The enzymatic reaction of GpdC prefers NADP^+^, which is distinct from GpdA that uses NAD^+^ as a cofactor, indicating that the NADP^+^-dependent GAPDH activity of GpdC tolerates RNS stress.

## Materials and methods

### Strains and growth conditions

We analyzed various strains of *Aspergillus nidulans* (Supplementary Table S1). Mitochondria in the SRS29 (Suelmann and Fischer, 2000) strain were assessed using fluorescence microscopy. *Aspergillus nidulans* strains were cultivated in Minimal Medium (MM; 10 g/L glucose, 6 g/L NaNO_3_ or 1.84 g/L ammonium tartrate, 0.52 g/L KCl, 0.52 g/L MgSO_4_·7H_2_O, 1.52 g/L KH_2_PO_4_, 1 mL/L Hutner’s trace element solution, pH 6.5). The pH of the medium was adjusted using 0.1 M NaOH. We added 0.05 mg/L pyridoxine, 1.22 g/L uracil +1.21 g/L uridine to the medium to cultivate the TN02A3 strain (Nayak et al., 2006). Sodium nitrite was added to sterilized medium, then the pH was adjusted to 5.5. Plasmids were manipulated and proteins were generated in *Escherichia coli* DH5α and BL21 (DE3) strains cultured in LB medium (5 g/L yeast extract, 10 g/L tryptone, 10 g/L NaCl).

### Construction of *Aspergillus nidulans* gene disruptants

The *gpdC* (Gene ID, AN2583), *pdcA* (AN4888), *alcC* (AN2286), and *dldA* (AN9066; *S. cerevisiae DLD1* ortholog) genes were replaced with the *pyrG* gene in *A. nidulans* TN02A3. We amplified DNA fragments encoding the 5′-and 3′-regions of the *gpdC*, *pdcA*, *alcC*, and *dldA* genes by PCR using the primer sets xxxX-FC/xxxX-R1, and xxxX-F3/xxxX-RC (“xxxX” represent gene names; Supplementary Table S2). Fragments of *pyrG* DNA were amplified by PCR using the primers pyrG-F and pyrG-R. The PCR template was genomic DNA of *A. nidulans* FGSC A4. Amplified fragments were fused by PCR using the primers xxxX-F1 and xxxX-R3, and transformed into *A. nidulans* TN02A3. Transformants were grown on MM agar. Gene disruption was confirmed by PCR using the primers xxxX-FC and xxxX-RC. Resulting disruptants were designated GpdCΔ, PdcAΔ, AlcCΔ, and DldAΔ (Supplementary Figure S1). The NPC1 strain constructed by introducing a *pyrG* gene fragment at the chromosomal *pyrG89* loci of the TN02A3 strain was the control (Supplementary Table S1).

### Introduction of *gpdC* to the GpdCΔ strain

We generated DNA fragments encoding the 5′-regions of *gpdC*, ORF of *gpdC*, and the 3′-region of *pyrG* by PCR using the primer sets; gpdC-FC/gpdCcomp-R1, gpdCcomp-F2/gpdCcomp-R2, and gpdCcomp-F4/gpdCcomp-RC, respectively (Supplementary Table S2). Genomic DNA of *A. nidulans* FGSC A4 was the template. The *ptrA* gene was a selection marker and corresponding DNA fragments were amplified using the primers gpdCcomp-F3/gpdCcomp-R3, and pPTRI (Kubodera et al., 2002). Resultant amplicons were fused by PCR using the primers gpdC-F1 and gpdCcomp-R4 (Supplementary Table S2), and transformed into the GpdCΔ strain. Transformants were cultivated on MM agar containing 0.1 μg/mL pyrithiamine. That *gpdC* was transduced into the target locus to generate the GpdCΔ+*gpdC* strain was confirmed by PCR using the primers gpdC-FC and gpdCcomp-RC (Supplementary Figure S2).

### Preparation of recombinant GpdA and GpdC

Conidia (2 × 10^7^) of the *A. nidulans* FGSC A4 strain were rotary shaken in 100 mL of MM at 60 rpm and 37°C for 14 h. Total RNA was extracted from powdered mycelia frozen in liquid nitrogen using RNeasy Plant Mini kits (Qiagen, Hilden, Germany), and cDNA was synthesized using PrimeScript RT Master Mix (Takara Bio, Shiga, Japan) as described by the manufacturer. Complementary DNA fragments for *gpdA* were generated by PCR using fungal cDNA and pET21a-gpdA-F and pET21a-gpdA-R primers (Supplementary Table S2). Complementary DNA fragments for *gpdC* (synthesized at Eurofins Tokyo, Japan) with optimized codons for *E. coli*, were digested by *Nde* I and *Hind* III, then cloned into pET-21a using Ligation High ver. 2 (Toyobo, Osaka, Japan).

Transformants harboring pET21a-gpdA and pET21a-gpdC were rotary-shaken in 3 mL of LB medium at 180 rpm at 37°C for 14 h, then 1 mL portions were inoculated into 100 mL LB medium, and rotary shaken at 160 rpm at 37°C. When the optical density reached 0.6, 0.5 mM isopropyl-*β*-D(−)-thiogalacto-pyranoside (IPTG) was added to the medium and incubated for 3 h. Harvested cells were resuspended and ultrasonicated in 10 mL of 20 mM HEPES-NaOH buffer (pH 7.5) containing 0.5 M NaCl and 20 mM imidazole, followed by centrifugation at 8,000 × *g* for 30 min. Supernatants were passed through a HisTrap FF crude column (Cytiva, North Logan, UT, United States). Thereafter, recombinant GpdA (rGpdA) and GpdC (rGpdC) were eluted with the same buffer containing 200 mM imidazole, and their homogeneity was confirmed by sodium dodecyl sulfate-polyacrylamide gel electrophoresis (SDS-PAGE).

### Measurement of GAPDH activities

We added 3 mM GAP to cuvettes containing rGpdA or rGpdC (10 μg each) in 90 μL of 1 mM NAD^+^, 10 mM potassium phosphate, 30 mM sodium pyrophosphate (pH 8.4), then changes in absorbance at 340 nm were monitored at 25°C for 2 min using a U-3900 spectrophotometer (Hitachi, Tokyo, Japan). The *K*_m_ and *k*_cat_ values were determined by using 0.01–1 mM NAD^+^, 0.01–10 mM NADP^+^, and 0.015–3 mM GAP. We incubated rGpdA and rGpdC (10 μg each) dissolved in 20 mM HEPES-NaOH buffer (pH 7.5) containing 150 mM NaCl) with NOC-5 and GSNO (Dojindo, Kumamoto, Japan) (0–1 mM each) at 25°C for 1 h, then we analyzed the susceptibility of GAPDH to NO donors.

### Quantitative real-time PCR

*Aspergillus nidulans* FGSC A4 conidia (2 × 10^7^) were inoculated into 100 mL of liquid MM, rotary shaken at 120 rpm and 37°C for 14 h, followed by 10 mM NaNO_2_, 3 mM H_2_O_2_, 50 μM menadione, 1.5 mM diamide, or 1 mM *tert*-butylhydroperoxide (*t*-BOOH) under the same conditions for 1 h. Mycelial cDNA was prepared as described in Section 1.4, then qRT-PCR proceeded using the THUNDERBIRD® SYBR® qPCR Mix (Toyobo), and the Thermal Cycler Dice Real-Time System MRQ (Takara Bio). Supplementary Table S2 shows the primer sequences.

### Measurement of intracellular GAPDH activity

*Aspergillus nidulans* NPC1 and GpdCΔ conidia (2 × 10^7^) inoculated into 100 mL of liquid MM (pH 5.5) were rotary shaken at 120 rpm and 37°C for 14  h, followed by 120 rpm and 37°C for 0–2  h after adding 10 mM NaNO_2_. Powdered mycelia (as described in Section 1.4) were dissolved in 1 mL of 20 mM Tris–HCl containing 150 mM NaCl/0.1 g mycelia, vortex-mixed, then centrifuged for 30  min at 18,800 × *g* and 4°C. We assessed GAPDH activity in the supernatant as described (Ralser et al., 2007) with the following modification. Supernatants (10 μL) were added to cuvettes containing 1 mM NAD^+^ in 90 μL of 30 mM sodium pyrophosphate (pH 8.4). The reaction was started by adding 3 mM GAP, then changes in absorbance at 340 nm were monitored at 25°C for 2 min (as described in section 1.5). We then determined the dry weight of lyophilized mycelia.

### Determination of cellular GAP and ethanol

*Aspergillus nidulans* conidia (2 × 10^7^) were cultured and mycelia were prepared as described in section 1.7. Ground mycelial powder (0.1 g) dissolved in 1 mL of 30 mM pyrophosphate buffer (pH 8.4) was vortex-mixed, and centrifuged for 30 min at 18,800 × *g* and 4°C. Intracellular GAP was quantified in supernatants as described by Garrigues et al. (1997) and modified as follows. Recombinant GpdA (50 μg) was added to cuvettes containing 100 μL of supernatants. Reactions were started by adding 1 mM NAD^+^, and changes in absorbance at 340 nm were monitored at 25°C for 5 min. Ethanol in culture media was quantified using F-kit ethanol (J.K. International, Tokyo, Japan) as described by the manufacturer.

### Biotin-switch assays

Recombinant GpdA and GpdC were incubated with 0–0.2 M GSNO at 25°C for 1 h, precipitated using an excess of acetone to remove GSNO, then resuspended in 100 μL of 20 mM HEPES-NaOH buffer (pH 7.5) containing 150 mM NaCl. Nitrosothiols were modified as described (Li and Kast, 2017) with the following modifications. Suspensions were mixed with equal volumes of 10 mg/mL *N*-ethylmaleimide, incubated at 37°C for 1 h, then proteins were precipitated using acetone. The precipitates were suspended in 100 μL of 20 mM HEPES-NaOH (pH 7.5) containing 150 mM NaCl, 1 mM biotin-HPDP solution (Dojindo) and 1 mM ascorbic acid, then incubated at 25°C for 1 h. Proteins were resolved by non-reducing SDS-PAGE, then *S*-nitrosothiol detected using horse radish peroxidase-conjugated streptavidin was analyzed by immunoblotting (Cosmo Bio, Tokyo, Japan).

### Phylogenetic analysis

Amino acid sequences of fungal GAPDH isozymes were downloaded from the Fungal and Oomycete genomics resource (FungiDB) database (https://fungidb.org/fungidb/app/). We analyzed their phylogenies using Molecular Evolutionary Genetics Analysis (MEGA) version 11.0.10 (Tamura et al., 2021) and the neighbor-joining method with complete gap deletion. Numbers and branches indicate values calculated from 1,000 bootstrap resampling replicates. The amino acid sequence of human GAPDH was obtained from the National Center for Biotechnology, and aligned with the fungal GAPDH using CLUSTALW version 2.1 (Larkin et al., 2007).

## Results

### Identification of RNS-inducible *gpdC*

**Figure 1:**
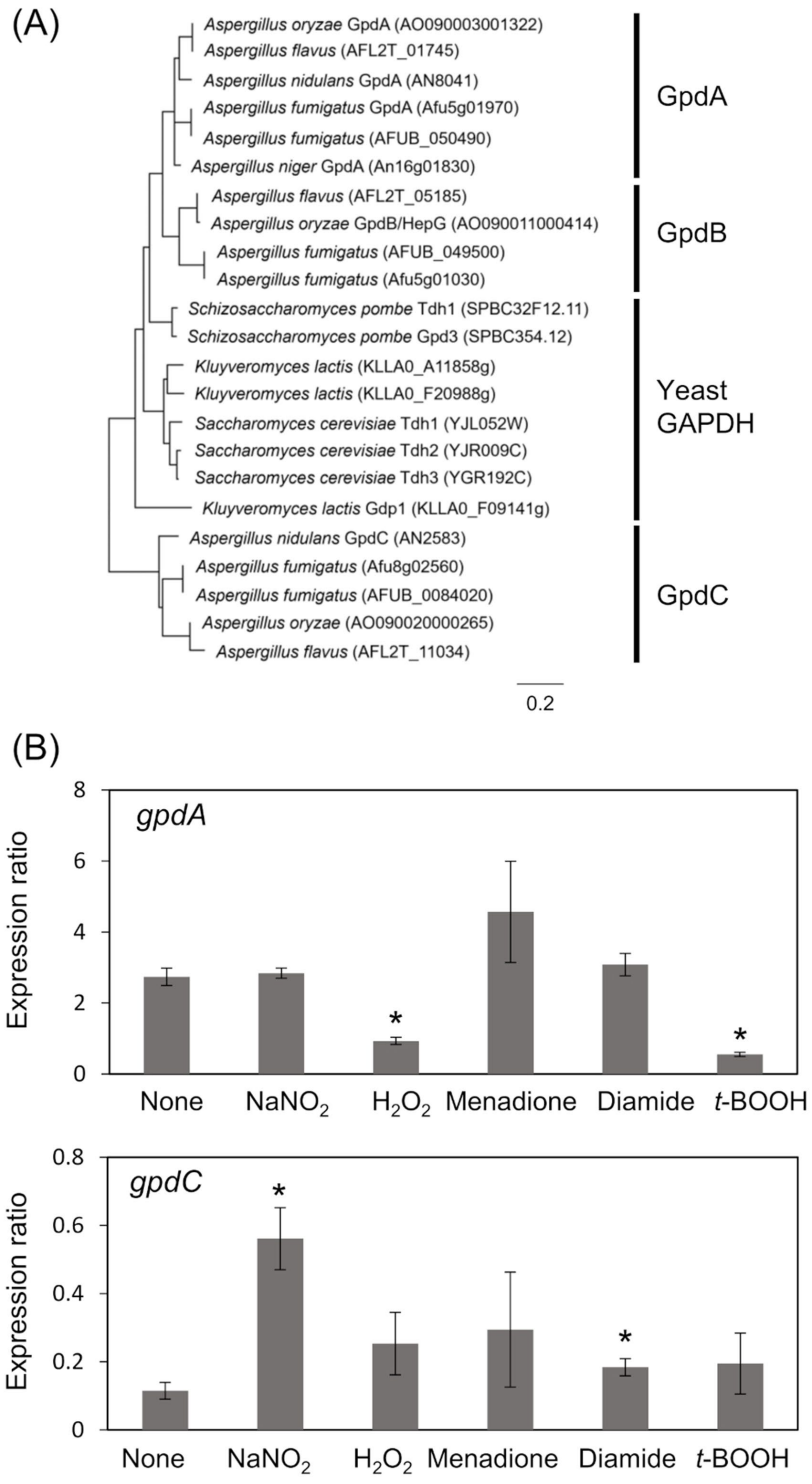
Phylogenetic analysis of Fungal GAPDH and transcription of GAPDH genes. (A) Phylogenetic tree of GAPDH in *Aspergillus* spp. and yeasts. Amino acid sequences were downloaded from FungiDB (https://fungidb.org/fungidb/app/) and phylogenetic tree was created using MEGA 11.0.10. (B) Mycelia of *A. nidulans* strain A4 were incubated at 37°C for 1 h with 10 mM NaNO_2_ (pH 5.5), 3 mM H_2_O_2_, 50 μM menadione, 1.5 mM diamide, and 1 mM *t*-BOOH. Amounts of transcripts were normalized to those of *Acta. means* and standard deviations were determined from results of three independent cultures. *p < 0.05 vs. non-stressed controls (Welch *t* test).

The *A. nidulans* genome consists of *gpdA* (Gene ID, AN8041) that encodes ubiquitous glycolytic GAPDH, and *gpdC* (AN2583) with unknown physiological function. The amino acid sequence identity between GpdA and predicted GpdC proteins was 47.0%. A phylogenetic analysis identified GpdA, B, and C isozymes in all *Aspergillus* species except *A. nidulans*, which lacks a gene encoding GpdB (Figure 1A). Here, we investigated the role of *A. nidulans* GpdC in nitrosative and oxidative stress responses because our transcriptome analysis suggested that *gpdC* expression is upregulated upon exposure to RNS (Amahisa et al., 2024). Transcripts of *gpdC* in *A. nidulans* mycelia were quantified by PCR after exposure to acidified nitrite (1 mM NaNO_2_, pH 5.5), H_2_O_2_, menadione, diamide, and *t*-BOOH as ROS donors for 1 h. We verified that acidified nitrite equilibrated with nitrosonium cations (NO^+^), imposes RNS stress on *A. nidulans* (Zhou et al., 2012). These results indicated that the fungus accumulated 4.9- and 1.6-fold more *gpdC* transcripts after exposure to acidified nitrite and diamide, respectively (Figure 1B). None of the other reagents altered the quantity of intracellular *gpdC* transcripts, whereas that of *gpdA* transcripts was decreased 0.34- and 0.20-fold by H_2_O_2_ and *t*-BOOH, respectively, but not by acidified nitrite. These results indicated that RNS stress induced *gpdC* expression.

### GpdC is a novel fungal NADP^+^- and NAD^+^-dependent GAPDH

**Figure 2:**
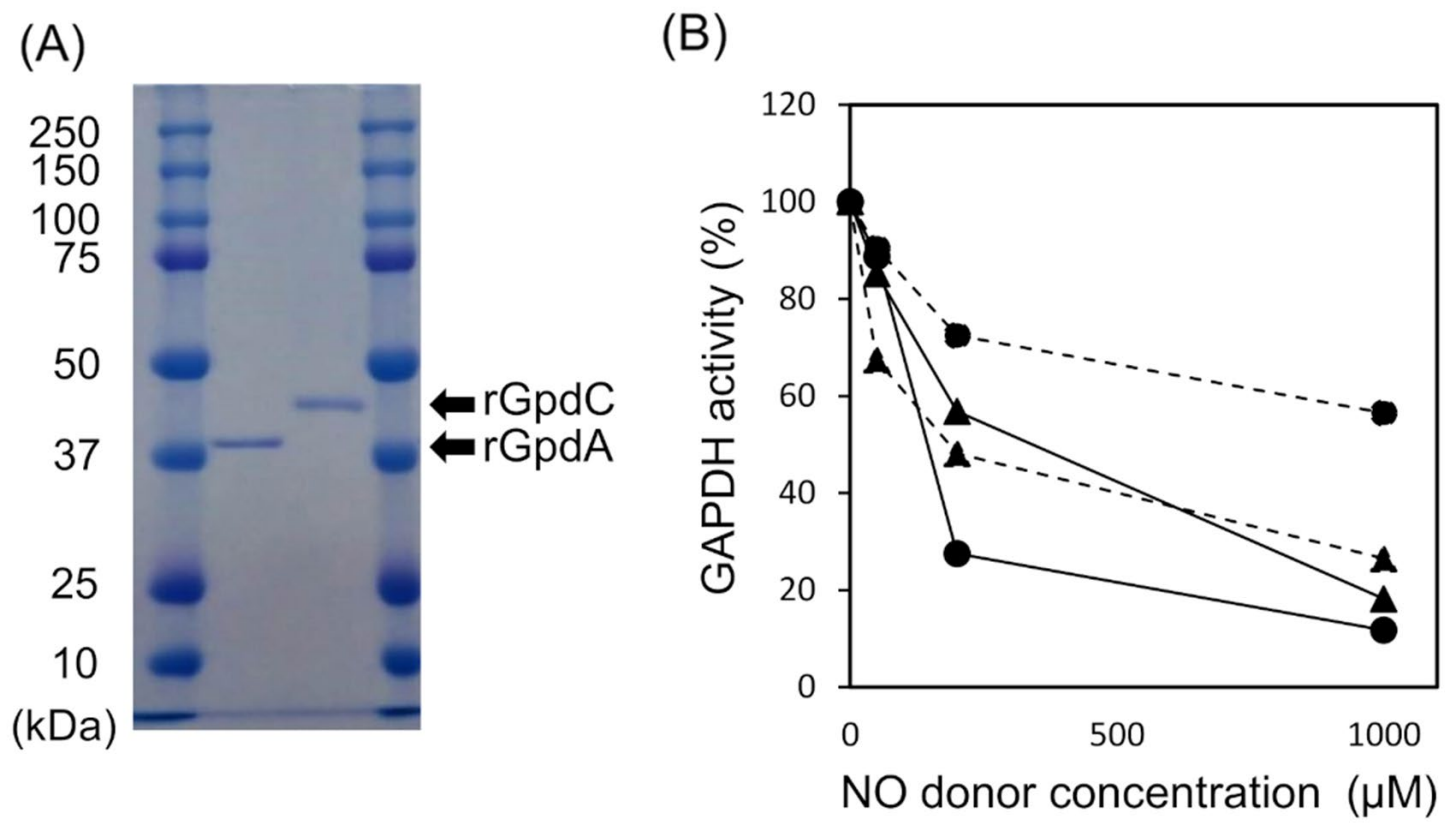
Properties of rGpdA and rGpdC. (A) Recombinant GpdA and GpdC proteins resolved by 12.5% SDS-PAGE. (B) GAPDH activities of GpdA (circles) and GpdC (triangles) after incubation with or without NOC-5 (*solid lines*) or GSNO (*dashed lines*) for 1 h. Values for GAPDH activity are relative to those incubated without NO donors. Cofactors for measuring rGpdA and rGpdC were NAD^+^ and NADP^+^ respectively.

Recombinant GpdA (rGpdA) and GpdC (rGpdC) were generated in *E. coli*. Purified proteins migrated as single bands on SDS-PAGE, indicating their homogeneity (Figure 2A). The specific activities of rGpdC on GAP-dependent NAD^+^ and NADP^+^ reduction were 4.1 ± 1.6 and 12 ± 2 μmol min^−1^ mg^−1^, respectively. The activities of rGpdA on GAP-dependent NAD^+^ and NADP^+^ reduction were 110 ± 10 and < 0.01 μmol min^−1^ mg^−1^, indicating that rGpdC and rGpdA oxidize GAP by using NADP^+^ and NAD^+^, and NAD^+^, respectively, as electron acceptors. Steady-state kinetics for the GAP-dependent NAD^+^- and NADP^+^-reduction were analyzed. The apparent Michaelis–Menten (*K*_m_) constant of the GpdC reaction for GAP was 290 ± 20 μM, which was similar to that of GpdA (Table 1). The *K*_m_ value for NAD^+^ was 420 ± 20 μM and comparable to that of rGpdA (410 ± 90 μM). These values were higher than that of NADP^+^ reduction by rGpdC (47 ± 2 μM), indicating that GpdC preferentially uses NADP^+^ over NAD^+^. These results indicated that GpdC is a novel NADP^+^-and NAD^+^-dependent GAPDH. The preference for NADP^+^ over NAD^+^ distinguished GpdC from GpdA, which has very low levels of NADP^+^-dependent GAPDH activity.

**Table 1:** Apparent kinetic parameters of rGpdA and rGpdC reactions.

Enzyme	Substrate	*K*_m_^app^ (μM)	*k*_cat_^app^ (min^−1^)	*k*_cat_^app^/*K*_m_^app^ (min^−1^ μM^−1^)
GpdA	GAP	410 ± 90	3,300 ± 200	7.9 ± 2.4
	NAD^+^	180 ± 30	5,100 ± 200	28 ± 7
	NADP^+^	NA	NA	NA
GpdC	GAP	290 ± 20	160 ± 20	0.55 ± 0.02
	NAD^+^	420 ± 20	240 ± 20	0.56 ± 0.02
	NADP^+^	47 ± 2	530 ± 30	11 ± 1

Initial velocity of NADH and NADPH generation was measured in 30 mM sodium pyrophosphate and 10 mM potassium phosphate (pH 8.4) at 25°C. NA, not analyzed due to enzyme activity below detection limit (0.01 μmol min^−1^ mg^−1^). Data are shown as means ± standard deviation (*n* = 3).

The enzymatic activity of mammalian GAPDH is susceptible to oxidative stress due to a ROS-labile catalytic thiol residue (Hara et al., 2006; Sirover, 2014) that reacts with the nitrosating reagent GSNO to generate *S-*nitrosothiol (Broniowska and Hogg, 2010). Amino acid sequence alignment showed that this thiol residue is conserved in GpdC (Supplementary Figure S4). We investigated the effects of NOC-5 (NO donor) and GSNO on the activities of the fungal GAPDH. After incubation at 25°C for 1 h, NOC-5 concentration-dependently decreased the activity of rGpdA and rGpdC (Figure 2B). At 0.2 mM, NOC-5 impaired the activity of rGpdA more than rGpdC, indicating that rGpdC is more resistant to NO. Adding GSNO also decreased GAPDH activity, but that of rGpdC was more susceptible. Biotin-switch methods visualized *S*-nitrosothiol of GSNO-treated rGpdA and rGpdC on blots, and this was elevated by increasing the GSNO concentration (Supplementary Figure S3). This confirmed that GSNO modified the thiol residues of rGpdA and rGpdC to *S*-nitrosothiol and impaired enzyme activity. The multiple alignment of GAPDH isozymes found five unique Cys residues in GpdC that were not found in human GAPDH and GpdA (Supplementary Figure S4). We consider that this finding complements the higher levels of nitrosation in GpdC (Supplementary Figure S3) because these residues provided extra thiol targets of the nitrosating reagents in GpdC.

**Figure 3:**
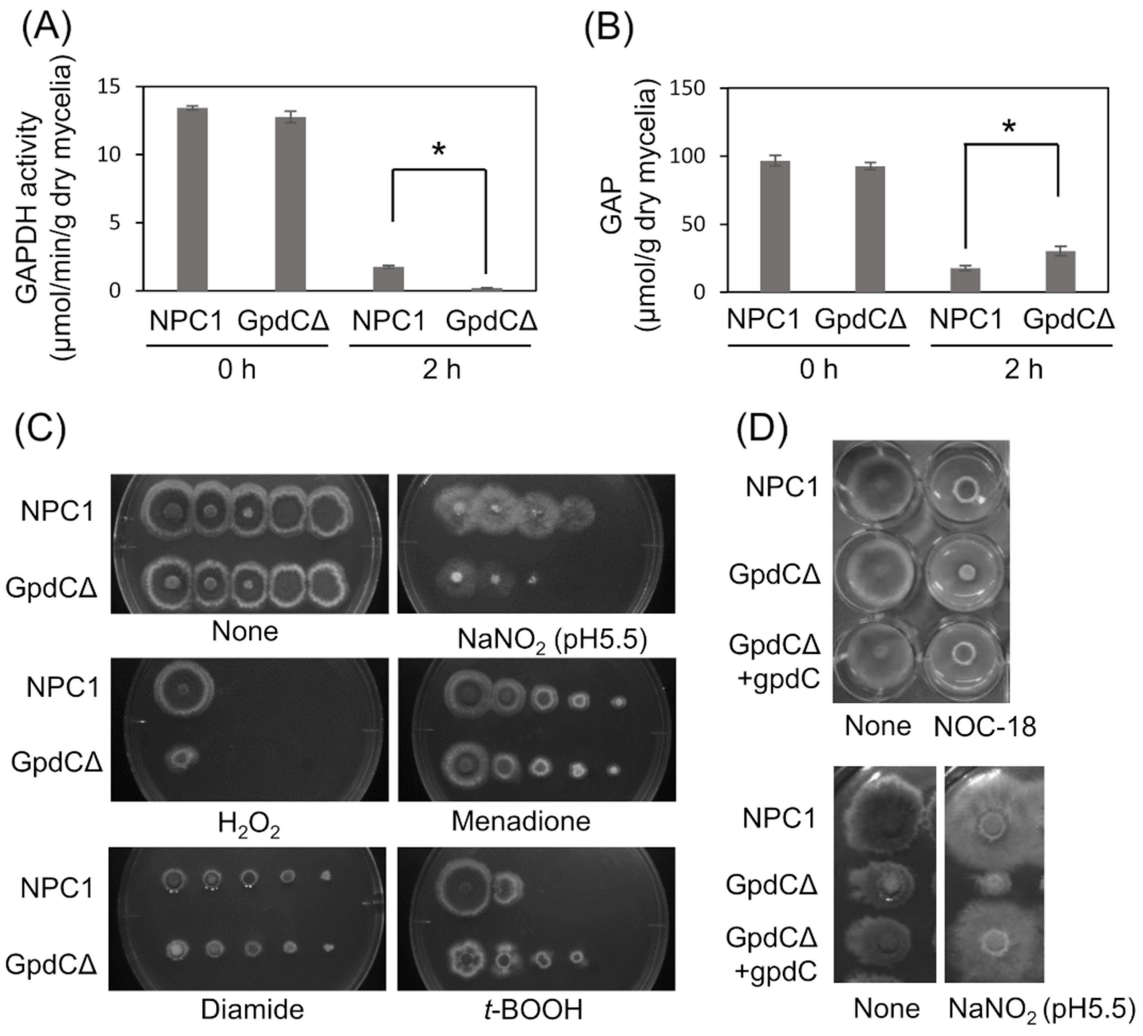
GpdC is required for fungal growth under RNS stress. (A) Activities of GAPDH produced by NPC1 and GpdCΔ strains incubated in MM for 14 h, followed by 0 and 2 h with 10 mM NaNO_2_. (B) Concentrations of GAP in NPC1 and GpdCΔ strains prepared as shown in panel *A. means* and standard deviations were determined from three independent cultures. *p < 0.05 (Welch *t* tests). (C) Strains NPC1 and GpdCΔ were incubated with MM agar (pH 5.5) with or without 30 mM NaNO_2_, 3 mM H_2_O_2_, 50 μM menadione, 1.5 mM diamide, and 1 mM *t*-BOOH at 37°C for 3 d. (D) Strains NPC1, GpdCΔ, and GpdCΔ +gpdC were incubated with MM agar (pH 5.5) supplemented with 10 mM NOC-18 and 30 mM NaNO_2_ at 37°C for 3 d. Assays including NOC-18 used NaNO_3_ as the nitrogen source and pH 6.5 was maintained in MM to avoid NOC-18 instability caused by pH decreases during culture. MM, minimal medium.

**Figure 4:**
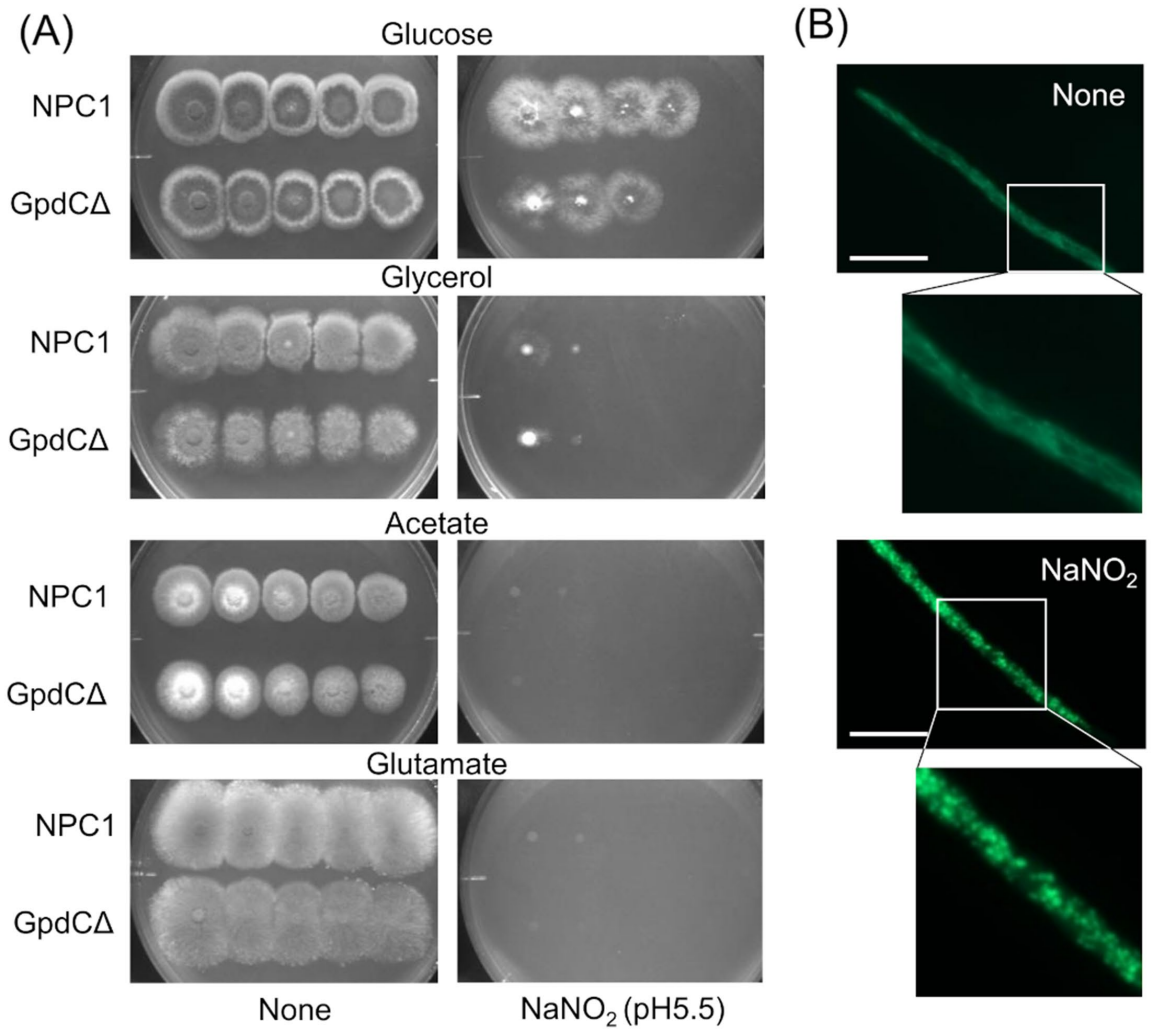
Growth of GpdCΔ using carbon sources and morphology of mitochondria under RNS stress. (A) Strains NPC1 and Δ*gpdC* were incubated on MM agar (pH 5.5) containing 1% glucose, 1% glycerol, 1% sodium acetate, or 1% sodium glutamate with or without 30 mM NaNO_2_ at 37°C for 3 d. (B) Mitochondria in strain SRS29 visualized by fluorescence microscopy of GFP. Scale bars, 10 μm.

### GpdC activity is required for growth under RNS stress

Intracellular GAPDH activity produced by the NPC1 (control *gpdC^+^*) strain and GpdCΔ that lacks intact *gpdC* (Supplementary Figure S1) were determined. Mycelia of the strains produced similar levels of activity in the absence of RNS stress, indicating that GpdC is not required for cellular GAPDH activity under these conditions (Figure 3A). This agreed with decreased *gpdC* expression in the absence of stress (Figure 2). Exposure to acidified nitrite (pH 5.5) for 2 h obviously decreased GAPDH activity, indicating impairment by RNS probably due to an oxidation-labile thiolate residue at the catalytic cysteine. This decrease was more prominent in the GpdCΔ strain that generated little GAPDH activity after exposure to acidified nitrite, which was consistent with the finding that this strain accumulated 1.7-fold more GAP than the NPC1 strain (Figure 3B). These results indicated that GpdC maintains cellular GAPDH activity, and that GpdC replaces RNS-sensitive GpdA under RNS stress.

The GpdCΔ strain grew slowly on agar containing acidified nitrite and H_2_O_2_ (Figure 3C). The growth rate was more rapid for the GpdCΔ, than the NPC1 strain in the presence of the NO donor diethylenetriamine (DETA)/NO adduct (NOC-18). Under these conditions, GpdCΔ produced a reddish brown pigment in the medium, which was not evident in the NPC1 strain (Figure 3D). Introducing *gpdC* into the GpdCΔ strain restored the growth rate of the strain (GpdCΔ + *gpdC*; Figure 3D), indicating that *gpdC* complemented the growth deficiency of the Δ*gpdC* strains. These results indicated that GpdC is a GAPDH isozyme, the activity of which confers *A. nidulans* growth tolerance under RNS stress.

### Reactive nitrogen stress promotes increased dependence on glycolytic metabolism

**Figure 5:**
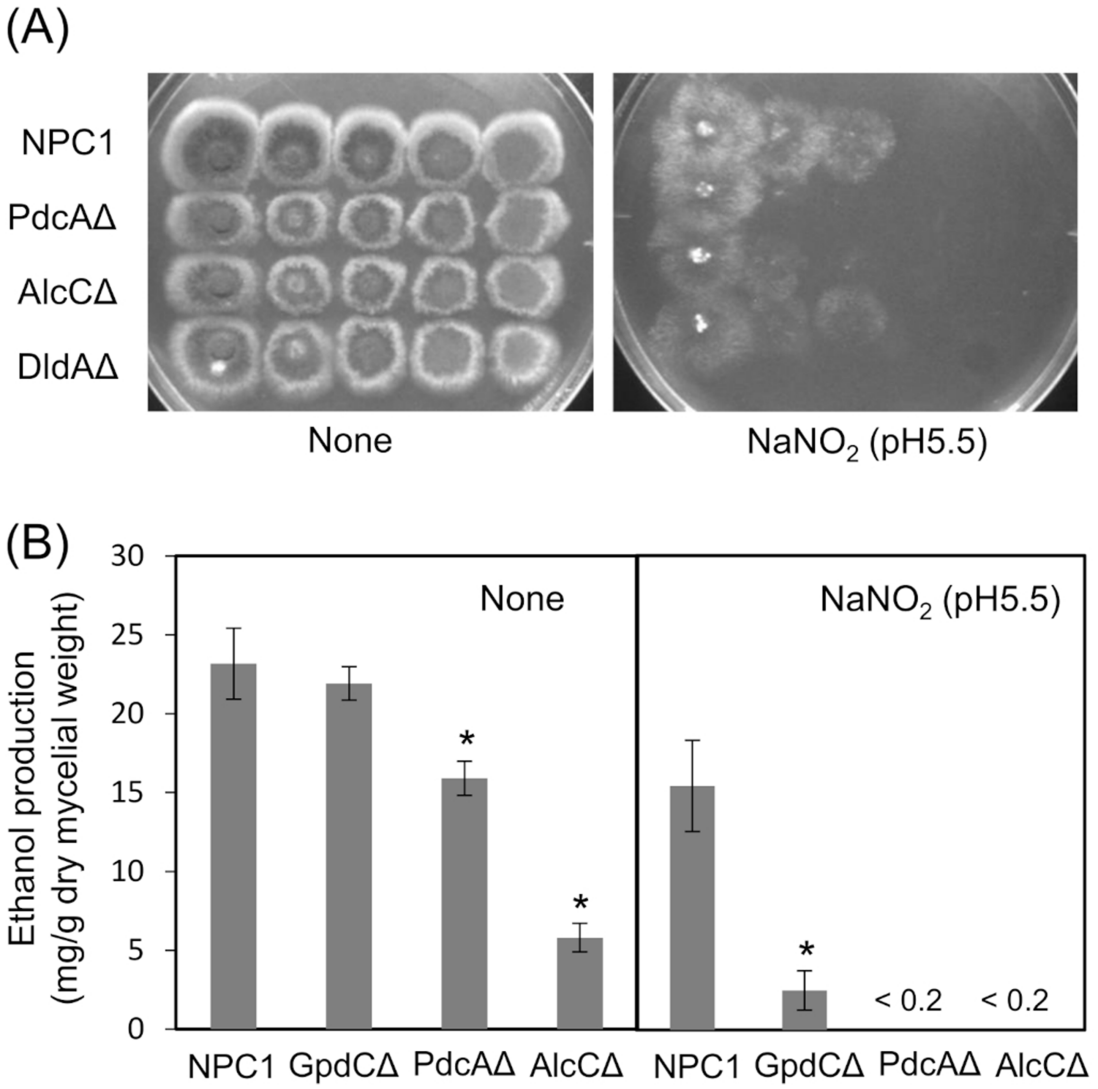
Glycolytic mechanism supports fungal growth under RNS stress. (A) Growth of NPC1, PdcAΔ, AlcCΔ and DldAΔ on MM agar (pH 5.5) supplemented with or without 30 mM NaNO_2_ at 37°C for 3. (B) Ethanol production by NPC1, GpdCΔ, PdcAΔ and AlcCΔ strains incubated in MM (pH 5.5) for 14 h followed by 12 h with 10 mM NaNO_2_. Means and standard deviations were determined from results of three independent cultures. *p < 0.05 vs. NPC1 (Welch *t* tests).

Reactive nitrogen species react with cellular thiols and metal ions, and mostly abrogate the respiration mechanisms of eukaryotic (Poderoso et al., 2019) and *A. nidulans* mitochondria (Amahisa et al., 2024; Zhou et al., 2012). These conditions might lead to increased dependence of the fungal growth on the glycolytic system. Figure 4A indicate that the acidified nitrite inhibited NPC1 growth more on agar media containing non-glycolytic glycerol, acetate, and glutamate as the carbon source compared with glucose. This suggested the importance of the glycolytic mechanism for fungal growth under RNS stress. The gene disruptants PdcAΔ (without *pdcA* encoding glycolytic pyruvate decarboxylase), AlcCΔ (*alcC*, alcohol dehydrogenase), and DldAΔ (*dldA*, putative lactate dehydrogenase) (Grahl et al., 2011) grew at comparable rates on medium containing glucose in the absence of RNS stress (Figure 5A). Acidified nitrite retarded the growth rates of PdcAΔ and AlcCΔ whereas these rates were comparable between DldAΔ and the control strain. These results indicated that the glycolytic ethanol fermentation mechanism is important for fungal growth under RNS stress.

Mitochondria were visualized using mitochondrial citrate synthase (CitA) fused to green fluorescent protein (GFP). Fluorescence shaped filaments, which are typical of fungal mitochondria under stress-free conditions (Figure 4B) dispersed into dot-like structures upon exposure to acidified nitrite. This dot-like morphology is also similar in fungal mitochondria under oxidative stress (Ruf et al., 2018). These results suggested that the RNS stress causes mitochondrial dysfunction and increased fungal metabolic dependence on glycolysis for proliferation.

### GpdC replaces GpdA in the glycolytic ethanol fermentation under RNS stress

We analyzed ethanol fermentation to understand the glycolytic role of GpdC under RNS stress. The NPC1 and GpdCΔ strains accumulated the same amounts of ethanol in culture media without acidified nitrite, whereas PdcAΔ and AlcCΔ accumulated 0.69- and 0.25-fold less ethanol than the NPC1 strain (Figure 5B). In contrast, GpdCΔ produced 0.16-fold more ethanol than the control strain, whereas PdcAΔ and AlcCΔ produced very little under RNS stress. Together with the defective growth under RNS stress (Figure 3), these results indicated that *A. nidulans* GpdC plays a significant role in energy conservation via ethanol fermentation as a mechanism to survive RNS stress.

**Figure 6:**
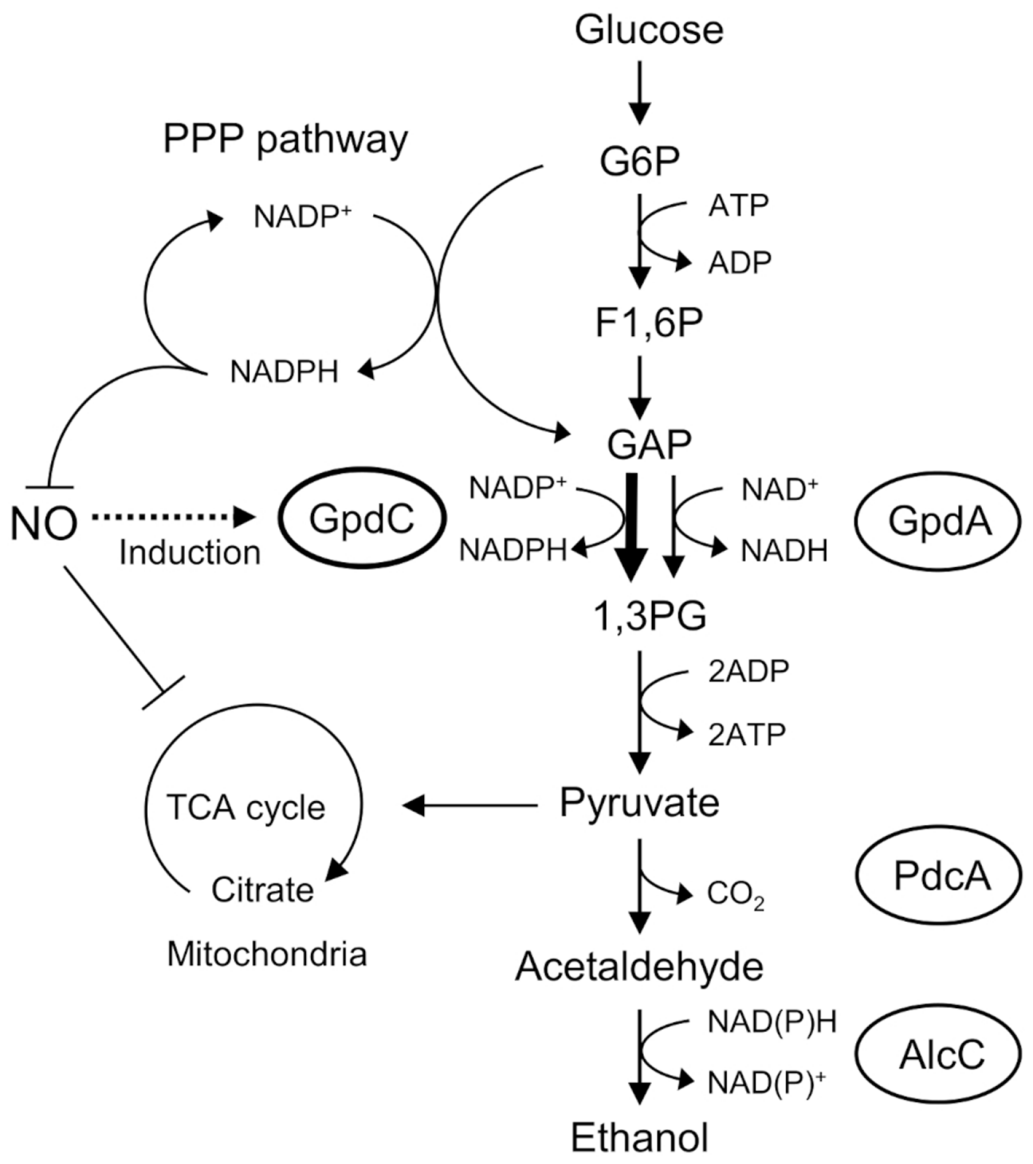
Mechanism of NO tolerance by GpdC in *A. nidulans*. F1, 6P, fructose 1,6-bisphosphate; GAP, glyceraldehyde 3-phosphate; G6P, glucose 6-phosphate; 1,3 PG, 1,3-*bis*-phosphoglycerate; PPP, pentose phosphate.

Figure 6 shows the mechanism of how GpdC renders confers fungal tolerance of RNS stress. The activity of canonical GpdA is largely impaired under RNS stress. *Aspergillus nidulans* induces expression of the *gpdC* gene and maintains intracellular levels of GAPDH activity. Increased tolerance against NO (Figure 2B) agrees with the notion that GpdC replaces GpdA. The NADP^+^-dependent activity of GpdC might contribute to maintain fungal glycolysis because NADPH production is associated with fungal oxidative and RNS stress responses (Yoshikawa et al., 2021). Thus, GpdC functions as an alternative to GpdA under RNS stress, whereas respiratory/mitochondrial defects increase the dependence of cellular energetic metabolism on a glycolytic mechanism.

## Discussion

This study uncovered a novel GAPDH, GpdC, that prefers NADP^+^ for catalysis. Plants and photosynthetic bacteria use NADP^+^-dependent GAPDH, which is involved in photosynthetic carbon fixation and gluconeogenesis, but not in glycolysis (Koksharova et al., 1998). Non-photosynthetic bacteria produce NAD^+^-and NADP^+^-dependent GAPDH to, respectively, facilitate glycolysis and gluconeogenesis (Fillinger et al., 2000). *Kluyveromyces lactis* Gdp1 was the first fungal NADP^+^-dependent GAPDH to be discovered, and it was thought to regenerate NADPH from NADP^+^ produced during xylose assimilation (Verho et al., 2002). The present study is the first to identify a glyceraldehyde 3-phosphate dehydrogenase GpdC as an NADP^+^-dependent GAPDH isozyme that is involved in the glycolytic mechanism and the ability of fungal cells to proliferate under RNS stress. This finding expands the diversity of GAPDH functions.

*Kluyveromyces lactis* Gdp1 is more similar to GpdA than GpdC (54.0% vs. 44.9% amino acid identity). A phylogenetic analysis indicated that it is closely related to GpdA (Figure 1A). A search of a published database showed that although GpdA-like GAPDH is widely distributed in fungi, GpdC-like GAPDH is found only in the fungal genera *Aspergillus*, *Botrytis*, and *Cryptococcus* (Flipphi et al., 2009). These genera include common plant and human pathogens. Considering that organisms develop functional orthologs under specific selection pressures, these fungi have acquired GpdC to survive the life-threatening environment of RNS. Nitric oxide produced by animal and plant NO synthases at sites of infected tissues can generate such an environment.

This study found that, GpdC loses more GAPDH activity than GpdA at lower GSNO concentrations (Figure 2B), indicating that tolerance against this level of RNS does not need GpdC to substitute the fungal glycolytic function of GpdA under RNS stress. The tolerance of the GpdC mechanism against RNS could be explained by its dependence on NADP^+^ activity. Under oxidative stress, metabolic flux shifts from the glycolytic, to the pentose phosphate pathway in *A. nidulans*, and increases intracellular levels of NAPDH (Ralser et al., 2007; Ralser et al., 2009; Talwar et al., 2023) that serves as a cofactor for antioxidant proteins, glutathione reductase (Sato et al., 2009), NO dioxygenase/flavohemoglobin, and thioredoxin reductase (Thön et al., 2007; Zhou et al., 2013). Their antioxidant reactions consequently generate NADP^+^. The NADP^+^-dependent GAPDH activity of GpdC enables *A. nidulans* to regenerate NADPH with concomitant glycolytic GAP oxidation, and hence conserves energy under RNS stress.

Mitochondrial dysfunction (Zhou et al., 2012; Amahisa et al., 2024) and disabled utilization of non-fermentable carbon sources by fungi exposed to RNS (Figure 5) evoke RNS stress that alters global cellular metabolism. We found here that RNS decreases the mitochondrial metabolic TCA cycle and amino acid biosynthesis (Amahisa et al., 2024), which concur with these findings. The fungus responds to RNS, induces the transcription of genes to synthesize amino acids via the master regulator CpcA, which is a counterpart of the yeast Gcn4p operating general amino acid control system, and maintains cellular amino acid synthesis (Amahisa et al., 2024). The finding that GpcC responded to RNS and maintained glycolysis adds another mechanism of metabolic regulation that allows fungi to adapt to RNS. This highlights the importance of metabolic regulation co-operating with RNS detoxification mechanisms mediated by specific proteins and factors (Frey et al., 2002; Liu et al., 2001; Zhou et al., 2013; Yoshikawa et al., 2023; Anam et al., 2020) to fungal responses and resistance to RNS.

Filamentous fungi, especially those in the genus *Aspergillus*, produce many industrially useful fermentation products such as organic acids and enzymes. Efficient production depends on appropriate control of cultures under oxidative and RNS stress. Infective *Aspergillus* pathogens are thought to counteract the RNS produced by NO produced by the host immune system. The discovery of GpdC involved in RNS tolerance might provide new targets to control these fermentation processes and antifungal agents to treat diseases.

## Data Availability

The original contributions presented in the study are included in the article/Supplementary material, further inquiries can be directed to the corresponding author/s.

## Supplementary material

### Supplementary Figure S1

Construction of A. nidulans gene disruptants. (A) Schematic representation of gene disruption. Arrows indicate positions and directions of primers (Supplementary Table S1). (B) Disruption of transformant genes confirmed by PCR.

### Supplementary Figure S2

Construction of gpdC and gpdC-complementation strains. (A) Schematic representation of gene disruption. Arrows indicate positions and directions of primers (Supplementary Table S1). (B) Disruption of transformant genes confirmed by PCR.

### Supplementary Figure S3

Detection of S-nitrosylated GAPDH. Upper panel: Biotin-switch assay of S-nitrosothiol 0, 1, 10, 50, 100, or 200 μM GSNO to 1 μg of purified GpdA and GpdC using streptavidin-conjugated horseradish peroxidase. Lower panel: protein detected using Ponceau S.

### Supplementary Figure S4

Multiple amino acid sequence alignment of GAPDH isozymes. Amino acid residues that were post-translationally modified in mammalian GAPDH are highlighted in light blue (nitrosation/oxidation), yellow (acetylation), and red (phosphorylation). Unique cysteine residues are boxed. Human GAPDH, NP_001276674.1; GpdA, AN8041; GpdC, AN2583.
