# Supplementary material for: Fungal glyceraldehyde 3-phosphate dehydrogenase GpdC maintains glycolytic mechanism against reactive nitrogen stress-induced damage
Source: Front Microbiol. 2024 Oct 11;15:1475567. doi: 10.3389/fmicb.2024.1475567 (PMC11502334; doi:10.3389/fmicb.2024.1475567)
Supplement: Supplementary file 1 [file Data_Sheet_1.PDF]

Supplementary Fig. S1 Kadooka et al.

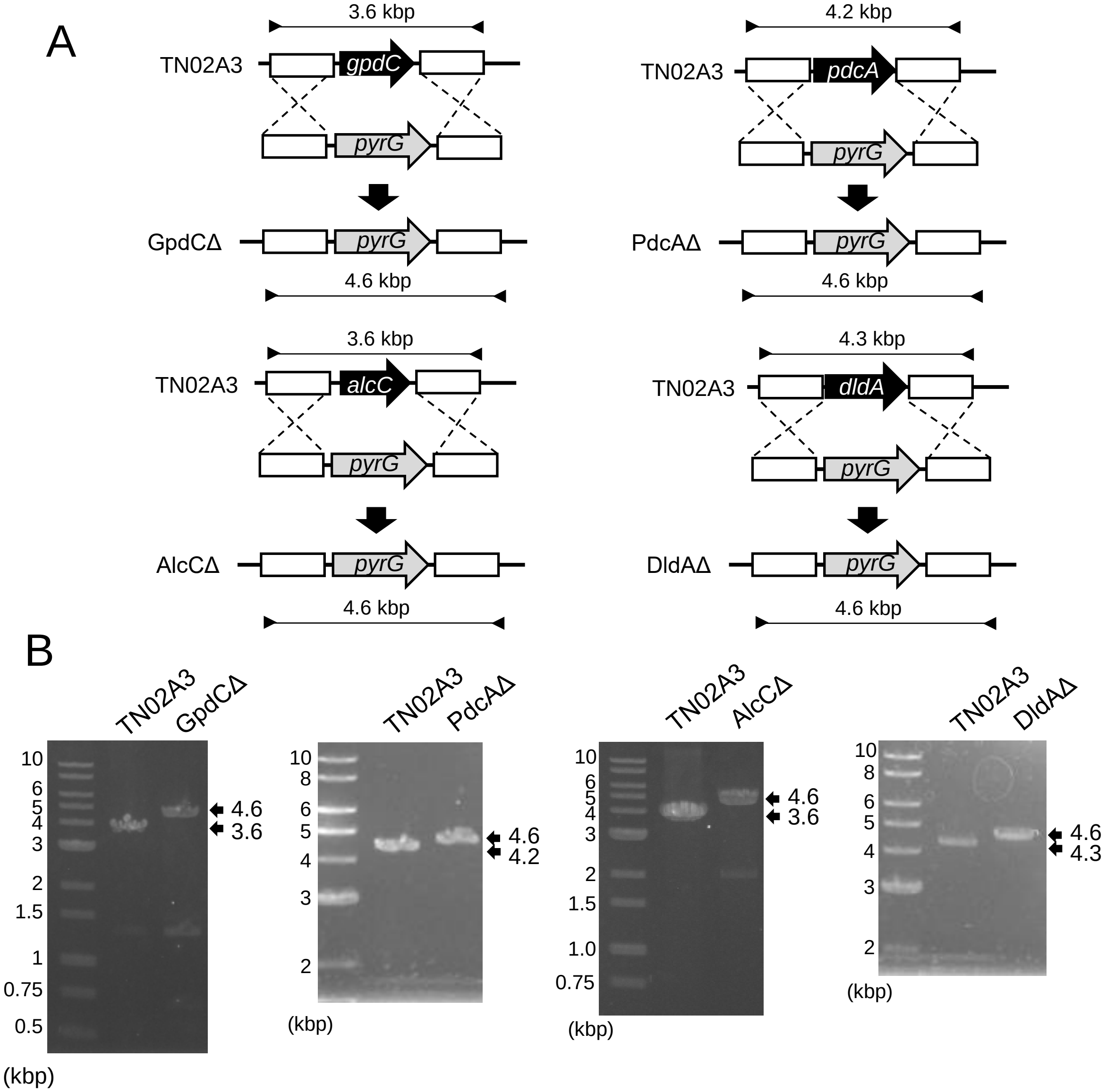

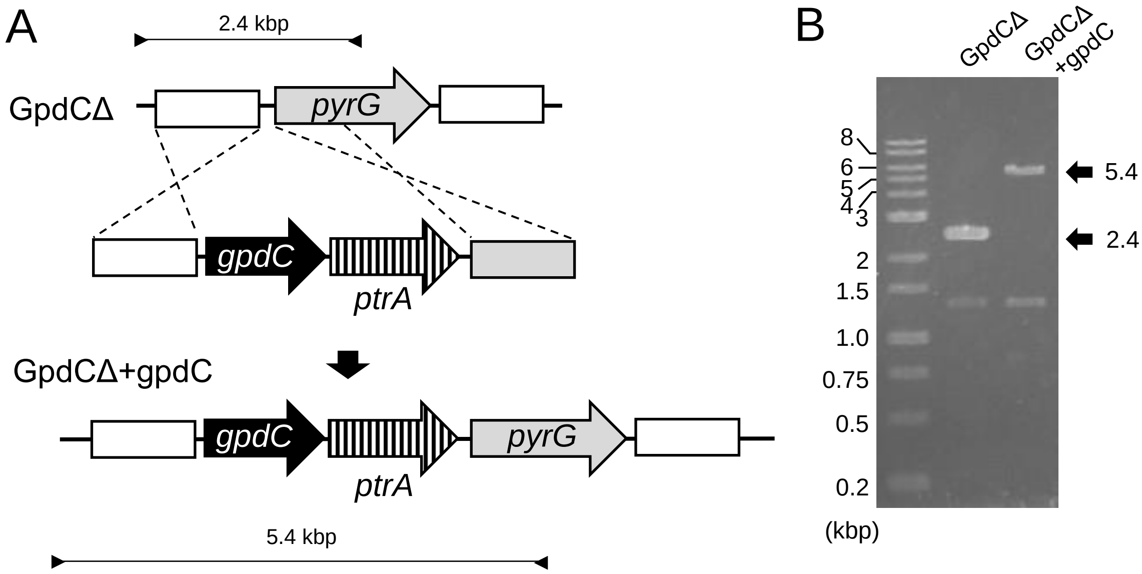
A
2.4 kbp
GpdCΔ
pyrG
gpdC
ptrA
GpdCΔ+gpdC
gpdC
ptrA
pyrG
5.4 kbp
B
GpdCΔ
GpdCΔ +gpdC
8
6
5
4
3
2
1.5
1.0
0.75
0.5
0.2
(kbp)
5.4
2.4

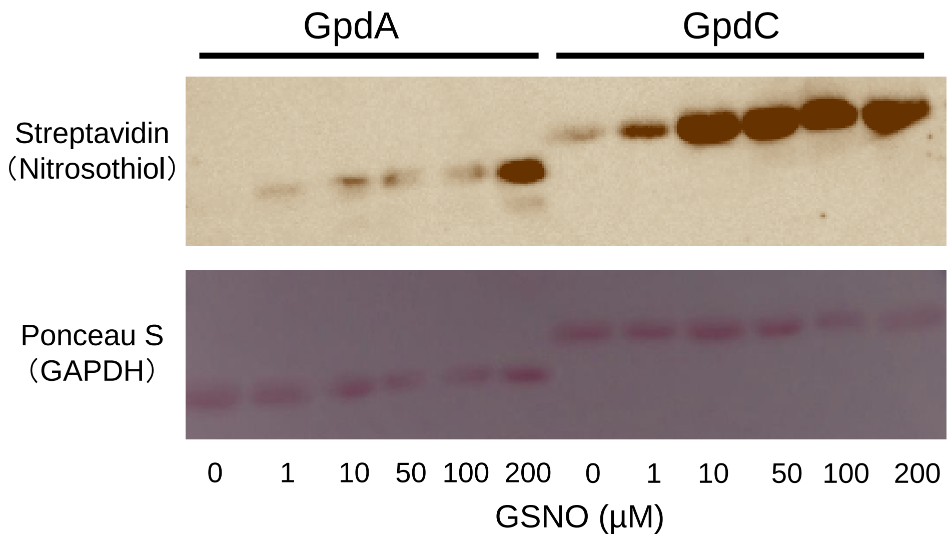
GpdA
GpdC
Streptavidin
(Nitrosothiol)
Ponceau S
(GAPDH)
0 1 10 50 100 200 0 1 10 50 100 200
GSNO (μM)

Supplementary Fig. S4 Kadooka et al.

```
Human GAPDH      -------------MGKVKVGVNGFGRIGRLVTRAAFNSGKVDIVAINDPFIDLNYMVYMF
GpdA             --------------MAPKVGINGFGRIGRIVFRNAIEAGTVDVVAVNDPFIETHYAAYML
GpdC             MAPSINEIPISDSPSACRVGINGFGRIGRNVLRVALARNDIQIVAINHTCTSVQDLIYLI
                                  :**:******** * * *:  . :::**:*..  . :   *::

Human GAPDH      QYDSTHGKFHGTVKAENG---KLVINGNPITIFQERDPSKIKWGDAGAEYVVESTGVFTT
GpdA             KYDSQHGQFKGTIETYDE---GLIVNGKKIRFHTERDPANIPWGQDGAEYIVESTGVFTT
GpdC             RYDSSMGKLSPDIPIHAISETQISVNGRPIALTSERDLKKLNWAALGAEYVVECTGKFTK
                 :***  *::   :         : :**. * :  ***  :: *.  ****:**.** **.

Human GAPDH      MEKAGAHLQGG-AKRVIISAPSADAPMFVMGVNHEKYDN--SLKIISNASCTTNCLAPLA
GpdA             QEKASAHLKGG-AKKVVISAPSADAPMFVMGVNNETYKK--DIQVLSNASCTTNCLAPLA
GpdC             RELAQDHITYGGAKRVVISAPSSDSPTYVYGVNADEYRANEETRVISCASCTTNCVTPVL
                  * *  *:  * **:*:*****:*:* :* *** : *    . :::* *******::*:

Human GAPDH      KVIHDNFGIVEGLMTTVHAITATQKTVDGPSGKLWRDGRGALQNIIPASTGAAKAVGKVI
GpdA             KVINDNFGIIEGLMTTVHSYTATQKVVDGPSAKDWRGGRTAATNIIPSSTGAAKAVGKVI
GpdC             KVLQREFGIAQGFLTTVHAATKSQTVLDGYSKKNRRLGRSVFDNIIPTTTGAAKAIASVL
                 **:: :*** :*::****: * :*..:** * *  * ** .  ****::******:..*:

Human GAPDH      PELNGKLTGMAFRVPTANVSVVDLTCRLEKPAKYDDIKKVVKQASEGPLKGILGYTEHQV
GpdA             PSLNGKLTGMAMRVPTSNVSVVDLTVRTEKAVTYDQIKDAVKKASENELKGILGYTEDDI
GpdC             PELTGKVTGASIRVPTPNVSLIDLTVSTETPTSLAEILAAFRRAAKNELAGVLSVSDEVL
                 *.*.**:** ::****.***::***   *....  :*  ..::*::. * *:*. ::. :

Human GAPDH      VSSDFNSDTHSSTFDAGAGIALNDHFVKLISWYDNEFGYSNRVVDLMAHMASKE--
GpdA             VSTDLNGDTRSSIFDAKAGIALNSNFIKLVSWYDNEWGYSRRVVDLITYISKVDAQ
GpdC             VSSDYLGDSHSAIVDAAACSELNPTFFKIMAWYDNEWGYSSRLLDLTRHIAHNE--
                 **:*  .*::*: .** *   **  *.*:::*****:*** *::**  :::  :
```

Nitrosation /Oxidation (blue) — Acetylation (yellow) — Phosphorylation (red)
